# The Cytoprotective Effect of Sulfuretin against *tert*-Butyl Hydroperoxide-Induced Hepatotoxicity through Nrf2/ARE and JNK/ERK MAPK-Mediated Heme Oxygenase-1 Expression

**DOI:** 10.3390/ijms15058863

**Published:** 2014-05-19

**Authors:** Dong-Sung Lee, Kyoung-Su Kim, Wonmin Ko, Bin Li, Gil-Saeng Jeong, Jun-Hyeog Jang, Hyuncheol Oh, Youn-Chul Kim

**Affiliations:** 1 Hanbang Body-Fluid Research Center, Wonkwang University, Iksan 570-749, Korea; E-Mail: hsds@wku.ac.kr; 2 College of Pharmacy, Wonkwang University, Iksan 570-749, Korea; E-Mails: pipo5@wku.ac.kr (K.-S.K.); rabis815@naver.com (W.K.); 3 Institute of Pharmaceutical Research and Development, College of Pharmacy, Wonkwang University, Iksan 570-749, Korea; 4 Department of Pharmacy, Qingdao University of Science & Technology, Qingdao 266042, China; E-Mail: leebin09@naver.com; 5 College of Pharmacy, Keimyung University, Dae-gu 704-701, Korea; E-Mail: gsjeong@kmu.ac.kr; 6 Department of Biochemistry, Inha University School of Medicine, Incheon 400-712, Korea; E-Mail: juhjang@inha.ac.kr

**Keywords:** sulfuretin, *Rhus verniciflua* Stokes, HepG2 cells, hepatoprotective, *tert*-butyl hydroperoxide, oxidative stress, heme oxygenase-1

## Abstract

Sulfuretin is one of the major flavonoid components in *Rhus verniciflua* Stokes (Anacardiaceae) isolates. In this study, we investigated the protective effects of sulfuretin against *tert*-butyl hydroperoxide (*t*-BHP)-induced oxidative injury. The results indicated that the addition of sulfuretin before *t*-BHP treatment significantly inhibited cytotoxicity and reactive oxygen species (ROS) production in human liver-derived HepG2 cells. Sulfuretin up-regulated the activity of the antioxidant enzyme heme oxygenase (HO)-1 via nuclear factor E2-related factor 2 (Nrf2) translocation into the nucleus and increased the promoter activity of the antioxidant response element (ARE). Moreover, sulfuretin exposure enhanced the phosphorylation of c-Jun *N*-terminal kinase (JNK) and extracellular signal-regulated kinase 1/2 (ERK1/2), which are members of the mitogen-activated protein kinase (MAPK) family. Furthermore, cell treatment with a JNK inhibitor (SP600125) and ERK inhibitor (PD98059) reduced sulfuretin-induced HO-1 expression and decreased its protective effects. Taken together, these results suggest that the protective effect of sulfuretin against *t*-BHP-induced oxidative damage in human liver-derived HepG2 cells is attributable to its ability to scavenge ROS and up-regulate the activity of HO-1 through the Nrf2/ARE and JNK/ERK signaling pathways. Therefore, sulfuretin could be advantageous as a bioactive source for the prevention of oxidative injury.

## 1. Introduction

Oxidative stress develops from an imbalance between the systems generating and scavenging reactive oxygen species (ROS). Excessive accumulation of ROS is involved in several human pathologies such as liver cirrhosis and fibrosis [1,2]. Antioxidants are considered therapeutic agents for counteracting liver damage, because oxidative stress plays a critical role in the pathology of liver diseases [3].

Heme oxygenase (HO) degrades heme to generate carbon monoxide (CO), free iron, and biliverdin [4]. HO-1, the inducible HO, is well known for its antioxidant and cytoprotective roles and expressed in response to oxidative stress [5]. Nuclear factor E2-related factor 2 (Nrf2), a regulator of the antioxidant response, plays a critical role in protecting cells against oxidative stress [6]. Under normal cell conditions, Nrf2 is inactivated in the cytoplasm through binding to its inhibitor protein, Kelch-like ECH-associated protein 1 (Keap1) [7]. The complex is disrupted by exposure to various stimuli, and free Nrf2 subsequently translocates into the nucleus and forms heterodimers with small Maf proteins and up regulates via interaction with antioxidant response element (ARE) [8,9]. This results in the induction of gene expression of phase II enzymes, including HO-1 [10]. Mitogen-activated protein kinases (MAPKs) play crucial roles in cellular processes, including proliferation, stress responses, apoptosis, and immune defense [11]. In general, *HO-1* gene expression is induced by stimuli that activate MAPKs [12,13]. MAPKs are classified into three major subgroups, including extracellular signal-regulated kinase (ERK), c-Jun *N*-terminal kinase (JNK), and p38 MAPK.

HepG2 cells, a human hepatoma cell line, are used to study *in vitro* metabolism and liver toxicity because they have many of the same functions as normal human hepatocytes [14]. Many studies use *tert*-butyl hydroperoxide (*t*-BHP) as the toxic agent to investigate the antioxidant effects in human liver-derived HepG2 cells [15,16]. *t*-BHP can be metabolized to free radicals by cytochrome P450 enzymes. These free radicals can initiate lipid peroxidation and mediate DNA damage, eventually causing cell death [17,18].

*Rhus verniciflua* (*R. verniciflua*) Stokes (Anacardiaceae) has been traditionally used as a food additive and herbal medicine in Korea and China [19]. Recent studies have shown that *R. verniciflua* has various biological activities, including antioxidant activity [20,21], antiobesity activity [22], anti-inflammatory effects [23], anti-mutagenic activity [24], and apoptotic effects, in human cancer cell lines [25–27]. Sulfuretin, the major flavonoid of *R. verniciflua*, is known to have various biological activities including anti-cancer [28], anti-inflammatory [29], anti-platelet [30], anti-mutagenic [24], and anti-rheumatoid arthritis [31] effects. However, to our knowledge, sulfuretin has not been previously reported to have a hepatoprotective effect. Therefore, we investigated the hepatoprotective effect of sulfuretin and the mechanism involved in this action in human liver-derived HepG2 cells stimulated with *t*-BHP.

## 2. Results and Discussion

### 2.1. Effects of Sulfuretin on the Viability of Human Liver-Derived HepG2 Cells

**Figure 1.**
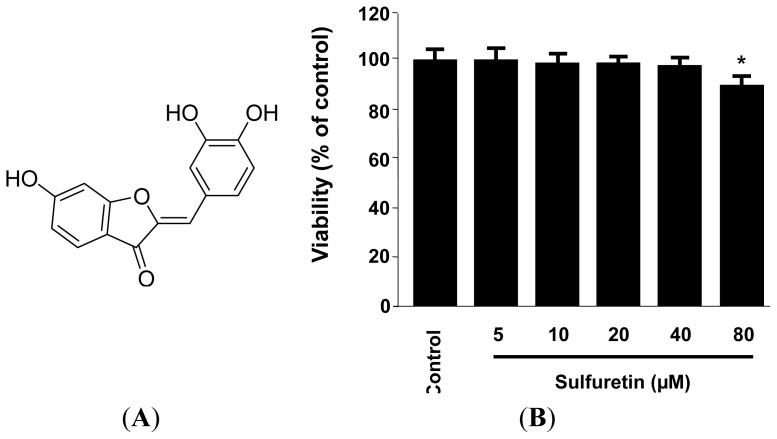
Chemical structure of sulfuretin (**A**) and effect of sulfuretin on the cell viability (**B**). Human liver-derived HepG2 cells were incubated for 24 h with various concentrations of sulfuretin (5–80 μM). Cell viability was determined by using MTT assay, as described in the Experimental section. Data shown represent the mean values of three experiments ± SD. * *p* < 0.05 *vs.* control.

We obtained sulfuretin in our previous study [32], and Figure 1A shows its structure. The cytotoxicity of sulfuretin on human liver-derived HepG2 cells was measured by using the MTT assay. As shown in Figure 1B, the viability of the cells incubated with varying concentrations of sulfuretin (5–40 μM) was not significantly affected; therefore, a concentration range of 5–40 μM was employed for all subsequent experiments.

### 2.2. Protective Effects of Sulfuretin on t-BHP-Induced Oxidative Injury and ROS Generation in Human Liver-Derived HepG2 Cells

**Figure 2.**
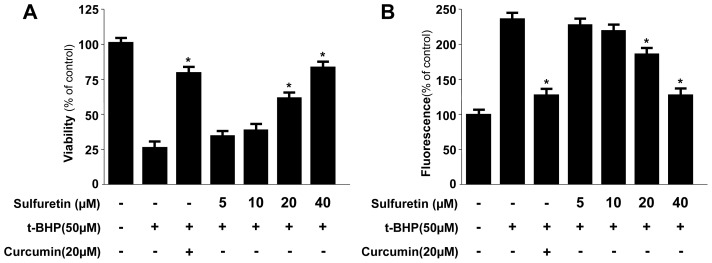
Protective effects of sulfuretin on *t*-butyl hydroperoxide-induced oxidative toxicity (**A**) and inhibition of ROS generation (**B**) in human liver-derived HepG2 cells. Cells were treated with sulfuretin and subsequently incubated for 12 h with *t*-butyl hydroperoxide (50 μM). Cell viability and ROS generation were determined as described in the Experimental Section. Data shown represent the mean values of three experiments ± SD. * *p* < 0.05 *vs. t*-BHP (50 μM) treatment.

It is well known that many liver diseases are associated with high levels of ROS and that oxidative protein and lipid modifications correlate with disease severity and progression. Therefore, regulating the generation of oxidative stress may be an important intervention in possible antioxidant therapy in liver diseases [1–3,33]. In this regard, natural compounds that possess antioxidant properties and can trigger intracellular cytoprotective signaling cascades in liver cells can be expected to serve as therapeutic agents. In this study, to test whether sulfuretin caused human liver-derived HepG2 cells to become more resistant to oxidative injury, the cells were exposed to *t*-BHP, a compound commonly used to cause oxidative stress in biological systems [33]. To test the protective effects of sulfuretin in human liver-derived HepG2 cells, the cells were pre-treated with sulfuretin (5–40 μM) for 12 h and were subsequently exposed for 12 h to *t*-BHP. At non-cytotoxic concentrations of sulfuretin (5–40 μM), pre-treatment of human liver-derived HepG2 cells with sulfuretin significantly protected at concentrations of 20 and 40 μM from *t*-BHP-induced oxidative cytotoxicity. *t*-BHP also significantly increased ROS production, and sulfuretin also effectively suppressed this action at concentrations of 20 and 40 μM (Figure 2). Curcumin, which is well known for its anti-oxidative and cytoprotective effects, was used as a positive control; this agent showed a significant cytoprotective effect and ROS scavenging activity at a concentration of 20 μM.

### 2.3. Effects of Sulfuretin-Induced HO-1 Expression on t-BHP-Induced Oxidative Injury and ROS Generation in Human Liver-Derived HepG2 Cells

The cytoprotective properties of antioxidants are commonly related to their ability to induce cytoprotective enzymes. HO-1, an enzyme essential for heme degradation, has been shown to exert anti-oxidative effects under various conditions [34]. The expression of HO-1 is considered an adaptive and protective response against oxidative insults in a wide variety of cells, including the HepG2 cell line [35]. In this respect, we have recently initiated our studies of the phytochemicals from natural products with interest in new pharmacological activities and mechanisms of the activities related to liver diseases involving HO-1 expression [36–38].

**Figure 3.**
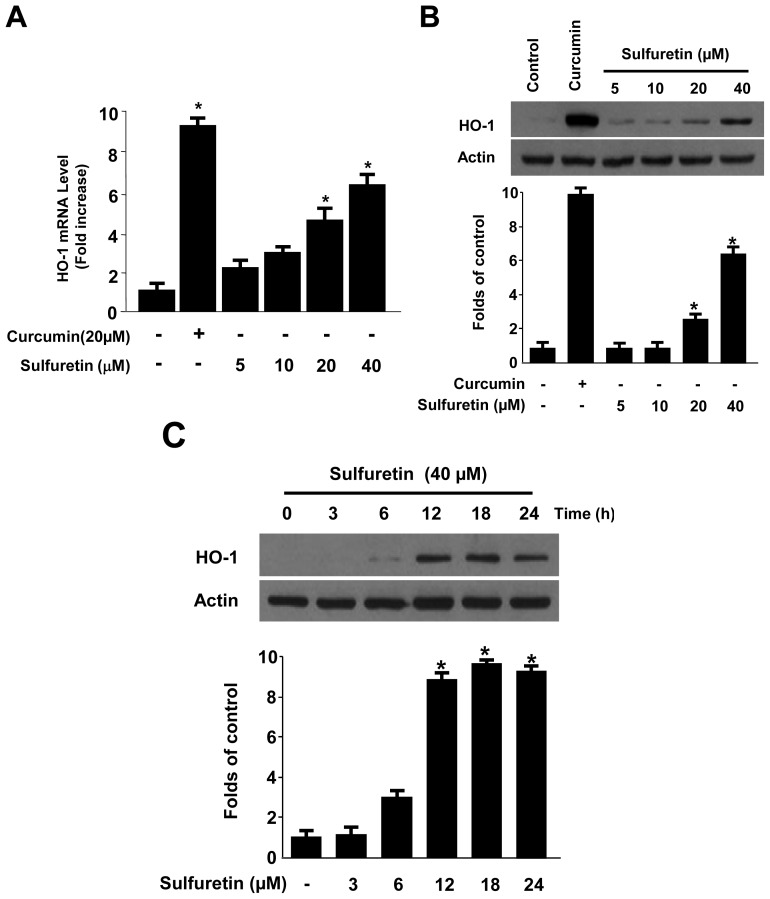
Effects of sulfuretin on heme oxygenase (HO)-1 mRNA (**A**) and protein (**B**,**C**) expression in human liver-derived HepG2 cells; (**A**,**B**) Cells were incubated with sulfuretin for 12 h; and (**C**) Cells were incubated with 40 μM of sulfuretin for the indicated periods. Data shown represent the mean values of three experiments ± SD. Expression of HO-1 was determined by western blot analysis, and representative blots from three independent experiments with similar results. * *p* < 0.05 *vs.* control.

**Figure 4.**
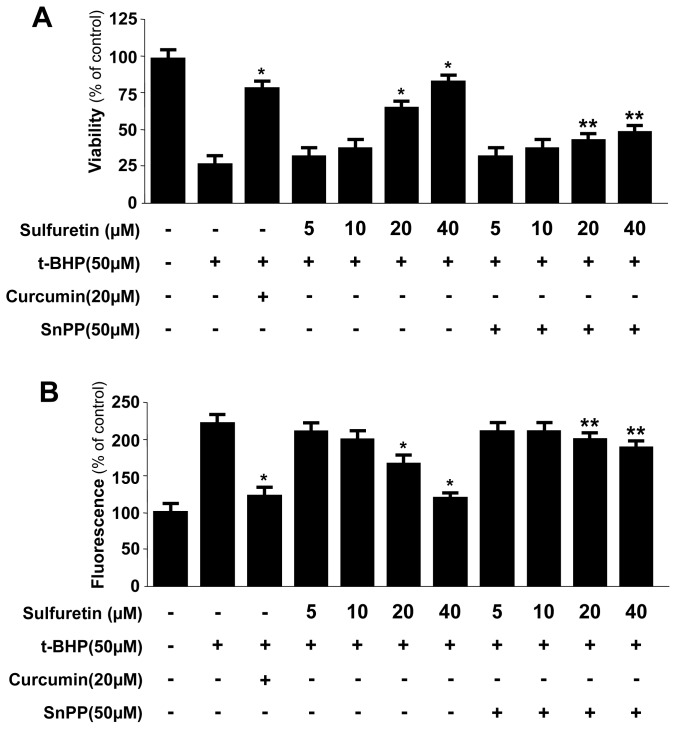
Effects of HO-1 Induction by sulfuretin on *t*-butyl hydroperoxide-induced oxidative toxicity (**A**) and ROS generation (**B**) in human liver-derived HepG2 cells. Cells were treated with various concentrations of sulfuretin and 50 μM tin protoporphyrin (SnPP), and were subsequently exposed to *t*-butyl hydroperoxide (50 μM) for 12 h. Cell viability and ROS generation were determined as described in the Experimental Section. Data shown represent the mean values of three experiments ± SD. * *p* < 0.05 *vs. t*-BHP (50 μM) treatment, ** *p* < 0.05 *vs.* same treatment plus SnPP.

Here, we investigated whether sulfuretin treatment affected HO-1 mRNA and protein expression in human liver-derived HepG2 cells. Treating the cells with various concentrations of sulfuretin (5–40 μM) for 12 h revealed that sulfuretin induced HO-1 mRNA and protein expression in a dose-dependent manner (Figure 3A,B). The natural product-derived HO-1 inducer curcumin, which was used as a positive control, increased HO-1 expression at 20 μM. HO-1 was induced after 6 h of treatment with a concentration of 40 μM, reached a maximum after 12 h, and then, it keeps constant at 24 h (Figure 3C). Next, we examined whether the induction of HO-1 expression by sulfuretin is involved in the protective effect and ROS-scavenging activity of sulfuretin. The cells were co-treated with 40 μM of sulfuretin for 12 h in the absence or presence of SnPP, a competitive inhibitor of HO activity. The competitive inhibitor of HO activity, SnPP, significantly suppressed sulfuretin-mediated cell protection and ROS deduction (Figure 4). These results suggested that HO-1 expression by sulfuretin is related to the protective effect and ROS-scavenging activity in HepG2 cells. In this result, SnPP partially reversed the ability of sulfuretin to suppress *t*-BHP-induced cytotoxicity and ROS generation (Figure 4). When cells are subjected to a variety of oxidative stresses, they typically respond by inducing a coordinated expression of genes encoding the set of phase II detoxifying enzymes, principally involved in activation of the transcription factor including Nrf2 [39]. The phase II detoxifying enzymes and related proteins play pivotal roles in protecting cells from free radical stress imposed by ROS. Key phase II detoxifying enzymes include HO-1, glutathione (GSH), glutathione-S-transferase (GST), NAD(P)H quinine oxido-reductase-1 (NQO1) and γ-glutamyl cysteine ligase (GCL), enhanced expression of which leads to an increase in levels of endogenous antioxidants such as the major thiol antioxidant GSH and reduced quinones [5,6,39]. The Nrf2-mediated regulation of antioxidant phase II detoxifying plays an important role in intracellular defense mechanisms. The focus of our study is on the cytoprotective effects of sulfuretin via up-regulation of HO-1 expression by Nrf2 signaling. In this study, we provided evidence to support the view that HO-1 expression, one of the key phase II detoxifying enzymes, through Nrf2 signaling pathways plays a key role in mediating the hepatoprotective effects of sulfuretin. Therefore, in our Figure 4, SnPP treatment and transient transfection partly attenuated the ability of sulfuretin to suppress *t*-BHP-induced ROS generation, meaning that the other phase II detoxifying enzymes as well as HO-1 may be involved in the mechanism of the hepatoprotective effects by sulfuretin.

### 2.4. Effects of Sulfuretin on HO-1 Expression through Nuclear Translocation of Nrf2 in Human Liver-Derived HepG2 Cells

Nrf2, a regulator of the anti-oxidant response, plays an important role in the transcriptional activation of the *HO-1* gene [40]. Recent studies have suggested that phytochemicals can activate Nrf2 by directly binding to Keap1 through covalent linkages, resulting in the induction of some cytoprotective proteins, such as HO-1 [41,42]. Furthermore, Nrf2 is translocated into the nucleus, whereupon it sequentially binds to the antioxidant response element (ARE) in the upstream promoter region of antioxidant phase II detoxifying enzymes [43]. Previous reports have shown that increasing Nrf2 activity in hepatic tissues is highly hepatoprotective against oxidative stress [44,45].

**Figure 5.**
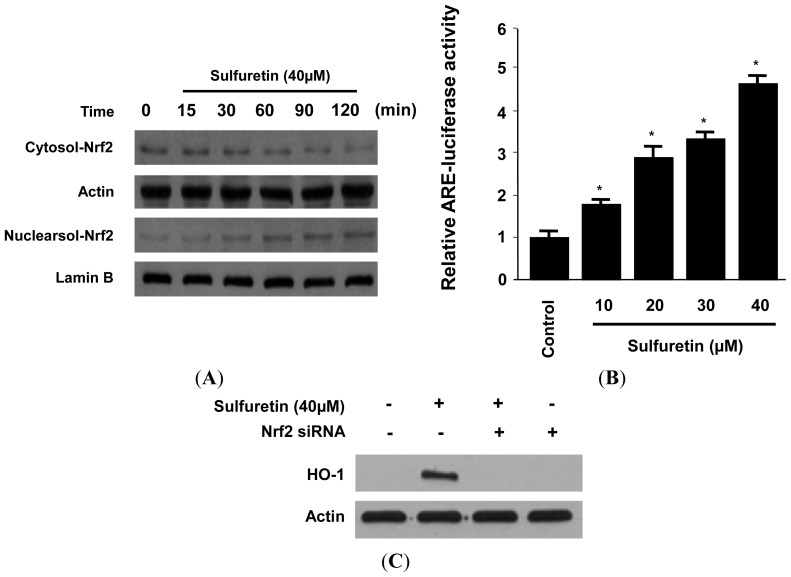
Effects of sulfuretin on Nrf2 nuclear translocation (**A**), antioxidant response element (ARE) activation (**B**), and transfection with Nrf2 siRNA (**C**) in human liver-derived HepG2 cells. (**A**) Cells were treated with 40 μM sulfuretin for 0–120 min. The nuclei were fractionated from the cytosol using PER Mammalian Protein Extraction Buffer as described in the Experimental section; (**B**) Quiescent cells transiently transfected with ARE-luciferase or control vector were incubated for 1 h with the indicated concentrations of sulfuretin in the presence of 5% fetal bovine serum (FBS). Cell lysates were assayed for the fold induction of luciferase activity by normalizing the transfection efficiency and dividing the values of each experiment relative to the control; and (**C**) Cells were transiently transfected with Nrf2 siRNA, and then treated with 40 μM of sulfuretin for 12 h. Nrf2 and HO-1 protein were detected by western blot analysis, and representative blots from three independent experiments with similar results. Data shown represent the mean values of three experiments ± SD. * *p* < 0.05 *vs.* control.

Therefore, we investigated whether treatment of human liver-derived HepG2 cells with sulfuretin induced nuclear translocation of Nrf2. When the cells were incubated with sulfuretin for 15–120 min at a concentration of 40 μM, this treatment resulted in a concomitant increase in the nuclear levels and a decrease in the cytoplasmic levels of Nrf2 (Figure 5A). In addition, human liver-derived HepG2 cells that were transiently transfected with the ARE-luciferase plasmid were exposed to sulfuretin, and changes in luciferase activity were used as a measure of ARE activation. The assay showed that sulfuretin increased ARE-driven luciferase activity in a dose-dependent manner (Figure 5B). In addition, the role of Nrf2 in HO-1 expression by sulfuretin was studied using siRNA against Nrf2. Human liver-derived HepG2 cells were transiently transfected with siRNA Nrf2, and were then treated with 40 μM sulfuretin for 12 h. As shown in Figure 5C, transient transfection with Nrf2 siRNA completely abolished HO-1 expression by sulfuretin. These suggest that sulfuretin-induced HO-1 expression occurs through the Nrf2/ARE signaling pathway in human liver-derived HepG2 cells.

### 2.5. Involvement of the MAPK Pathways in Sulfuretin-Induced HO-1 Expression

**Figure 6.**
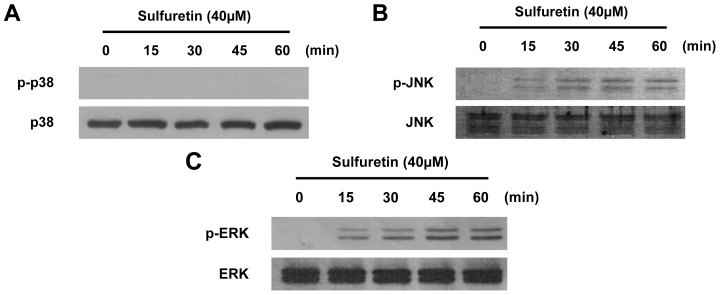
Effects of sulfuretin on MAPKs activation in human liver-derived HepG2 cells. Cells were treated with 40 μM sulfuretin for the indicated times (**A**–**C**). Cells were incubated with 40 μM of sulfuretin for the indicated periods. Activation of p38 (**A**); JNK (**B**) and ERK1/2 (**C**) were determined by western blot analysis. Membranes were stripped and re-probed for the total amount of each MAPK antibody as a control, and representative blots from three independent experiments with similar results.

**Figure 7.**
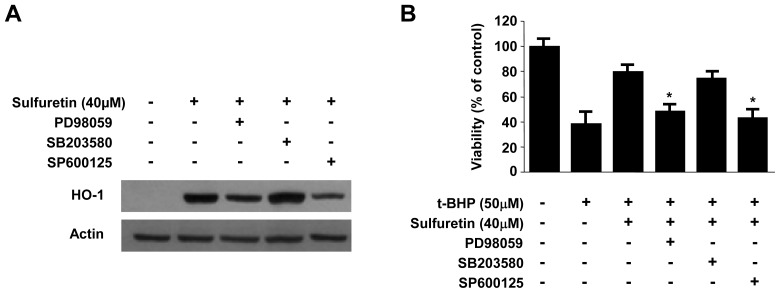
Effects of sulfuretin-induced mitogen-activated protein kinases (MAPKs) activation on HO-1 expression (**A**) and *t*-BHP-induced cytotoxicity (**B**) in human liver-derived HepG2 cells. Cells were incubated with 40 μM of sulfuretin for 12 h in the presence or absence of SP600125 (25 μM), SB203580 (20 μM), or PD98059 (10 μM). Then, cells subsequently were exposed to 50 μM of *t*-BHP for 12 h. Representative blots from three independent experiments with similar results. Data shown represent the mean values of three experiments ± SD. * *p* < 0.05 *vs. t*-BHP (50 μM) treatment.

Furthermore, activation of the JNK and ERK pathways appeared to be involved in sulfuretin-induced HO-1 expression (Figures 6 and 7). Several reports have shown that HO-1 expression is induced by the activation of MAPKs [46,47]. Under oxidative conditions, up-stream regulators of the Nrf2 cascade such as ERK1/2, JNK, and p38 play crucial role for activation of this cascade. In addition, the inhibition of MAPKs leads to a decrease in ARE-dependent gene expressions [48]. Therefore, we examined the effect of sulfuretin on the activation of MAPKs in human liver-derived HepG2 cells. At a concentration of 40 μM, sulfuretin activated the JNK and ERK kinase pathways and increased JNK and ERK phosphorylation in human liver-derived HepG2 cells. Phosphorylation of JNK and ERK kinases was observed at 15 min after sulfuretin treatment and was sustained up to 60 min after sulfuretin treatment (Figure 6). In contrast, phosphorylation of p38 kinases was not observed at any time point. Furthermore, to investigate the role of MAPK in HO-1 expression in human liver-derived HepG2 cells, we examined the effects of specific inhibitors of p38 kinases (SB203580, 4-(4′-fluorophenyl)-2-(4′-methylsulfinylphenyl)-5-(4′-pyridyl)imidazole), JNK (SP600125, 1,9-pyrazoloanth-rone), and ERK (PD98059, 2′-amino-3′-methoxyflavone) on the expression level of HO-1 by western blot analysis. We found that sulfuretin-induced HO-1 expression was inhibited by the JNK and ERK kinase inhibitors, whereas p38 kinases inhibitors had no effect (Figure 7A). In addition, cell viability was partially decreased when sulfuretin treatment was combined with JNK and ERK inhibitors in human liver-derived HepG2 cells (Figure 7B). These investigations showed that sulfuretin induced HO-1 expression via activation of JNK, ERK and Nrf2–ARE signaling in human liver-derived HepG2 cells.

## 3. Experimental Section

### 3.1. Materials

The heartwood of *R. verniciflua* Stokes (Anacardiaceae) was purchased from Dongbu Market, Iksan, Korea, in March 2009. The voucher specimen (WK-2009-42) was deposited at the Herbarium of College of Pharmacy, Wonkwang University (Iksan, Korea). Sulfuretin (Figure 1A) was isolated from the heartwood of *R. verniciflua* Stokes (Anacardiaceae) by using a previously described method [32]. RPMI 1640, fetal bovine serum (FBS), and other tissue culture reagents were purchased from Gibco BRL Co., (Carlsbad, CA, USA). Tin protoporphyrin IX (SnPP IX), an inhibitor of HO activity, was obtained from Porphyrin Products (Logan, UT, USA). All other chemicals were obtained from Sigma Chemical Co., (St. Louis, MO, USA) unless otherwise indicated. The human liver-derived HepG2 cells were obtained from the American Type Culture Collection (Manassas, VA, USA). The cells were maintained at 5 × 10^5^ cells/mL in RPMI 1640 medium supplemented with 10% heat-inactivated FBS, penicillin G (100 units/mL), streptomycin (100 mg/mL), and L-glutamine (2 mM), and were incubated at 37 °C in a humidified atmosphere of 5% CO_2_ and 95% air. Primary antibodies, including HO-1, Nrf2, lamin B, and actin, and secondary antibodies used for western blot analysis were purchased from Santa Cruz Biotechnology (Santa Cruz, CA, USA).

### 3.2. Cell Viability Assay

For determination of cell viability, 50 mg/mL of 3-[4,5-dimethylthiazol-2-yl]-2,5-diphenyltetrazolium bromide (MTT) was added to 1 mL of cell suspension (1 × 10^5^ cells/mL in 96-well plates) following by a 4 h incubation period. The formazan formed was dissolved in acidic 2-propanol, and the optical density was measured at 590 nm. The optical density of formazan formed in control (untreated) cells was regarded as 100% viability.

### 3.3. Preparation of Nuclear and Cytosolic Fractions

Human liver-derived HepG2 cells were homogenized in PER-Mammalian Protein Extraction Buffer (1:20, *w*/*v*) (Pierce Biotechnology, Rockford, IL, USA) containing freshly added protease inhibitor cocktail I (EMD Biosciences, San Diego, CA, USA) and 1 mM phenylmethanesulfonyl fluoride (PMSF). The cytosolic fraction of the cells was prepared by centrifugation at 15,000× *g* for 10 min at 4 °C. Nuclear and cytoplasmic extracts of cells were prepared using NE-PER nuclear and cytoplasmic extraction reagents (Pierce Biotechnology, Rockford, IL, USA), respectively.

### 3.4. Western Blot Analysis

Human liver-derived HepG2 cellswere harvested and pelleted by centrifugation at 200× *g* for 3 min. Then, the cells were washed with PBS and lysed in 20 mM Tris-HCl buffer (pH 7.4) containing a protease inhibitor mixture (0.1 mM PMSF, 5 mg/mL aprotinin, 5 mg/mL pepstatin A, and 1 mg/mL chymostatin). Protein concentration was determined using a Lowry protein assay kit (Sigma Chemical Co., St. Louis, MO, USA). Thirty microgram of protein from each sample was resolved by 12% sodium dodecyl sulfate-polyacrylamide gel electrophoresis (SDS-PAGE), and then electrophoretically transferred onto a Hybond enhanced chemiluminescence (ECL) nitrocellulose membrane (Bio-Rad, Hercules, CA, USA). The membrane was blocked with 5% skimmed milk and sequentially incubated with the primary antibody from Santa Cruz Biotechnology (Santa Cruz, CA, USA) and Cell Signaling Technology (Cell Signaling, MA, USA) and a horseradish peroxidase-conjugated secondary antibody followed by ECL detection (Amersham Pharmacia Biotech, Piscataway, NJ, USA).

### 3.5. ROS Measurement

Human liver-derived HepG2 cells (2.5 × 10^4^ cells/mL in 24-well plates) were treated with 50 μM of *t*-BHP in the presence or absence of test compounds or SnPP, a HO inhibitor, and were incubated for 8 h. After washing with PBS, the cells were stained with 10 mM 2′,7′-dichlorofluorescein diacetate (DCFDA) in Hanks’ balanced salt solution for 30 min in the dark. The cells were washed twice with PBS and were extracted with 1% Triton X-100 in PBS for 10 min at 37 °C. Fluorescence was recorded at an excitation wavelength of 490 nm and an emission wavelength of 525 nm (SpectraMax Gemini XS; Molecular Devices, Sunnyvale, CA, USA).

### 3.6. ARE Promoter Activity

To construct the ARE-luciferase vector, tandem repeats of double-stranded oligonucleotides spanning the Nrf2 binding site (5′-TGACTCAGCA-3′) were introduced into the restriction sites of the pGL2 promoter plasmid (Promega, Madison, WI, USA). All transfection experiments were performed by using Lipofectamine reagent (Invitrogen, Carlsbad, CA, USA) according to the manufacturer’s instructions. For luciferase assays, the cell lysate was first mixed with the luciferase substrate solution (Promega, Madison, WI, USA), and luciferase activity was measured by using an AutoLumat LB953 luminometer (EG and G Berthold, Bad Wildbad, Germany). For each experiment, the luciferase activity was determined in triplicate and was normalized in each sample by using β-galactosidase activity.

### 3.7. Real-Time PCR

Total RNA was isolated from the cells by using Trizol (Invitrogen), in accordance with the manufacturer’s recommendations, and quantified spectrophotometrically (at 260 nm). Total RNA (1 μg) was reverse transcribed using the High Capacity RNA-to-cDNA kit (Applied Biosystems, Carlsbad, CA, USA). The cDNA was then amplified using the SYBR Premix Ex Taq kit (TaKaRa Bio Inc., Shiga, Japan) by using a StepOnePlus Real-Time PCR system (Applied Biosystems, Carlsbad, CA, USA). Briefly, each 20 μL of reaction volume contained 10 μL of SYBR Green PCR Master Mix, 0.8 μM of each primer, and diethyl pyrocarbonate (DEPC)-treated water. The primer sequences were designed using PrimerQuest (Integrated DNA Technologies, Cambridge, MA, USA). The primer sequences were as follows: HO-1, forward 5′-CTCTTGGCTGGCTTCCTT-3′, reverse 5′-GGCTCCTTCCTCCTTTCC-3′, and GAPDH, forward 5′-ACTTTGGTATCGTGGAAGGACT-3′, reverse 5′-GTAGAGGCAGGGATGATGTTCT-3′. The optimum conditions for PCR amplification of the cDNA were established by following the manufacturer’s instructions. The data were analyzed using StepOne software (Applied Biosystems, Carlsbad, CA, USA), and the cycle number at the linear amplification threshold (*C*_t_) values for the endogenous control gene (glyceraldehyde 3-phosphate dehydrogenase (GAPDH)) and the target gene were recorded. Relative gene expression (target gene expression normalized to the expression of the endogenous control gene) was calculated using the comparative *C*_t_ method (2^−ΔΔ^*^C^*^t^).

### 3.8. Transfection

The cells were transiently transfected with Nrf2 siRNA for 6 h by using LipofectAMINE 2000™ (Invitrogen, Carlsbad, CA, USA), according to the manufacturer’s protocol, and recovered in fresh media containing 10% FBS for 24 h.

### 3.9. Statistical Analysis

Data were expressed as the mean ± SD of at least three independent experiments. To compare three or more groups, one-way analysis of variance followed by the Newman-Keuls post hoc test was used. Statistical analysis was performed with GraphPad Prism software, version 3.03 (GraphPad Software Inc., San Diego, CA, USA).

## 4. Conclusions

Recently, the tendency to use phytochemicals derived from plants to treat various diseases has increased because natural products are regarded as an important source for drug discovery and development [49,50]. The present study showed that sulfuretin isolated from *R. verniciflua* induced HO-1 expression through Nrf2/ARE and JNK/ERK-dependent pathways in human liver-derived HepG2 cells, leading to the inhibition of *t*-BHP-induced oxidative cell death and ROS production. Our study indicates that sulfuretin may be a potential bioactive agent for the prevention of oxidative injuries.
